# No Correlation Between Interferon Signaling and Cytosolic Mitochondrial DNA/RNA Leakage in Cultured Skin Fibroblasts of Patients With Mitochondrial Diseases

**DOI:** 10.1002/eji.70176

**Published:** 2026-03-27

**Authors:** Manon Marchais, Alessandra Pennisi, Alexandre Pierga, Alice Lepelley, Nicolas Cagnard, Christine Bole, Patrick Nitschke, Mohamed Hamici, Frédéric Rieux‐Laucat, Manuel Schiff, Arnold Munnich, Agnès Rötig

**Affiliations:** ^1^ Institut Imagine, Genetics of Mitochondrial Diseases, INSERM UMR 1163 Université Paris Cité Paris France; ^2^ Institut Imagine, Neurogenetics and Neuroinflammation, INSERM UMR 1163 Université Paris Cité Paris France; ^3^ Institut Imagine, Bioinformatic Platform Université Paris Cité Paris France; ^4^ Institut Imagine, Genomics Platform Université Paris Cité Paris France; ^5^ Institut Imagine, Immunogenetics of Pediatric Autoimmune Diseases, INSERM UMR 1163 Université Paris Cité Paris France; ^6^ Service et Centre de Référence des Maladies Héréditaires du Métabolisme AP‐HP, Hôpital Necker‐Enfants Malades Paris France; ^7^ Centre de Référence des Maladies Mitochondriales CARAMMEL AP‐HP, Hôpital Necker‐Enfants Malades and Imagine Institute Paris Paris France

## Abstract

Mitochondria have long been known to be involved in the regulation of innate immune response. We questioned whether cultured skin fibroblasts of patients suffering from mitochondrial diseases are valuable biological resources for the study of interferon signaling. Expression of interferon‐stimulated genes was measured in control cells supplemented with interferon and in cultured fibroblasts of patients carrying pathogenic variants in mitochondrial disease‐causing genes. Control fibroblasts showed a strong expression of interferon‐stimulated genes in response to interferon, but only 43% of patients’ fibroblasts displayed increased interferon stimulated genes scores. Cytosolic mitochondrial DNA and RNA were quantified by immunofluorescence and confocal microscopy. No correlation between elevated interferon response and cytosolic mitochondrial DNA or RNA release could be established. We found that cultured skin fibroblasts represent a valuable biological resource for the investigation of interferon signaling, but that abnormal interferon signaling is not always observed in patients with mitochondrial diseases. At variance to gene silencing in control fibroblasts, the lack of correlation between elevated interferon response and cytosolic mitochondrial DNA or RNA leakage in patients’ fibroblasts questions the relevance of cellular models as illustrators of pathological situations in humans.

## Graphical abstract

Investigation of IFN signaling in cultured skin fibroblasts from patients with genetically confirmed mitochondrial diseases of diverse origins revealed that altered IFN signaling is an inconsistent feature of these disorders. Cytosolic accumulation of mtDNA and mtRNA was variably detected and showed little correlation with ISG scores.

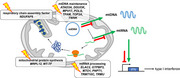

## 1 Introduction

Mitochondria have long been recognized as an important proxy of response to pathogenic agents and inflammation. When mitochondrial nucleic acids are released in the cytosol from damaged mitochondria, they activate the type I interferon (IFN) reaction, probably due to their similarity with bacterial nucleic acids. Experimental models have shown that defects in mitochondrial DNA (mtDNA) maintenance, replication, transcription, or RNA processing—such as *Tfam* gene heterozygous deletion—downregulation of human mitochondrial polynucleotide phosphorylase PNPase or ATAD3A lead to cytosolic accumulation of mtDNA or mitochondrial RNA (mtRNA) and exhibited a type I IFN response ascribed to activation of the cGAS promoting STING‐TBK1‐IRF3‐dependent IFN signaling pathway by released mtDNA [1, 2, 3]. More recently, human fumarase deficiency has been shown to induce the release of mtDNA in the cytosol, also triggering an inflammatory response [4]. Fumarase inhibition can also induce the release of mitochondrial RNA (mtRNA) and further IFN‐β production [5], and itaconate, a metabolite with immunomodulatory properties, has been shown to inhibit SDH, leading to double‐stranded mtRNA release [6].

Mitochondrial diseases are heterogeneous disorders caused by impaired oxidative phosphorylation. They are characterized by a wide range of clinical presentations, and more than 300 nuclear and mitochondrial disease genes have been hitherto reported [7]. Although immune abnormalities are rarely described, chronic type I IFN upregulation has been reported in patients with pathogenic variants in genes involved in mtDNA maintenance, mtRNA processing, and mitochondrial protein synthesis and presenting with Aicardi‐Goutières‐like syndrome, cystic leukoencephalopathy mimicking CMV infection, systemic sclerosis, sideroblastic anemia and immunodeficiency or interferonopathy and carrying pathogenic variants in mitochondrial protein coding genes, namely *PNPT1* [8], *RNASET2* [9, 10], *ATAD3A* [3], *TRNT1* [11], or *REXO2* [12], respectively.

Along the same lines, an increase of cell‐free mtDNA has been found in the serum of mitochondrial encephalomyopathy, lactic acidosis, and stroke‐like episodes (MELAS) patients carrying various mtDNA pathogenic variants with activation of the cGAS‐STING pathway in their muscle [13]. Similarly, analysis of peripheral blood from Friedreich ataxia patients [14] and other mitochondrial diseases [15] revealed an increased inflammatory signaling gene expression, including type I IFN, interleukin‐1β, and antiviral responses. Recently, Keshavan et al. [16] found upregulation of *IFI27* (encoding the interferon alpha‐inducible protein 27) but not of other IFN‐stimulated genes (ISG) in a wide range of primary and secondary mitochondrial diseases in blood.

Using a large series of cultured skin fibroblasts from patients carrying pathogenic variants in genes involved in various mitochondrial functions, we questioned whether cultured skin fibroblasts are suitable models to study IFN signaling and whether increased IFN response is a constant feature in mitochondrial diseases.

## 2 Results

### 2.1 Transcriptomic Analysis of Control Fibroblasts in Response to Type I IFN

**FIGURE 1:**
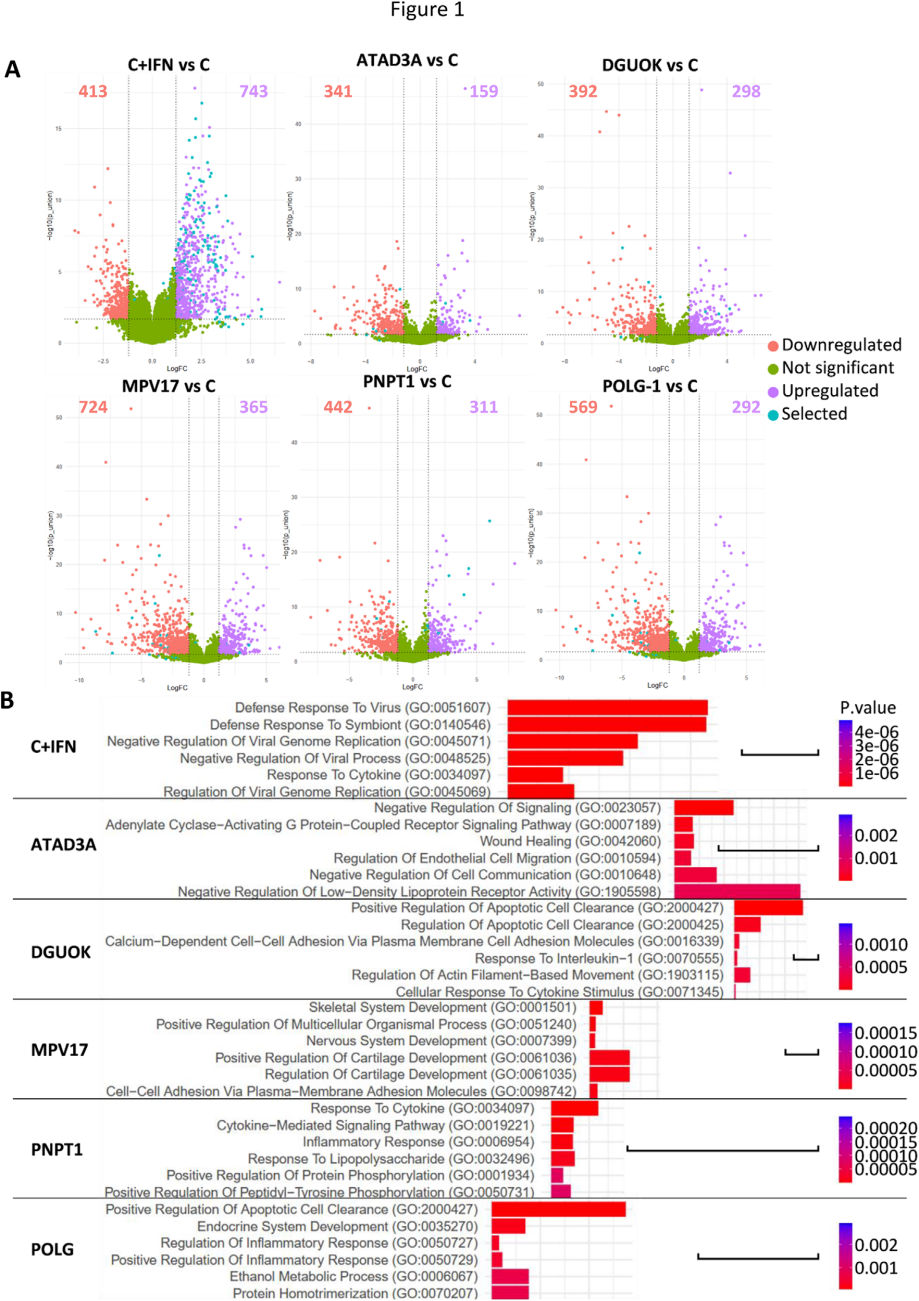
Transcriptomic analysis. **(A)** Volcano plots of differentially expressed genes in IFN‐treated control fibroblasts (C+IFN) and patients’ fibroblasts vs control fibroblasts (C). Down‐ and upregulated genes and their numbers are shown in red and purple, respectively. Unchanged genes are in green. Cyan dots indicate genes previously reported as ISGs(17). Results were considered statistically significant for *p*‐values of ≤0.05 and a fold change of ≥2.3. **(B)** Functional enrichment analysis by GO Biological Process of the different fibroblasts vs control fibroblasts. Bar scale: combined score of 500.

Exposure of control fibroblasts to IFNα for 6 h induced a robust transcriptional response. RNAseq analysis showed that 23.16% of mRNAs (743/3208) were significantly upregulated, with strong enrichment of IFN‐related pathways (genes with Log_2_FC>1.2, *p*‐value < 0.002, Figure 1A). Among the upregulated genes, several have been reported to be ISGs [17] (Figure 1A, cyan dots), and nearly half of them (369, 49%) were found in the interferome database [18] (Table S1). Gene Ontology (GO) terms, KEGG, and Reactome pathway analyses showed a strong enrichment of genes involved in IFN signaling in treated fibroblasts (Figure 1B; Figure S1). Further query for co‐occurrence of the “interferon” eponym with the remaining gene symbol citation databases found that 31 additional genes were reportedly upregulated by IFN or were known to regulate IFN (Table S1). Among the 62 most upregulated genes (>10‐fold change), 6 were not reportedly induced by IFN (*LINC01629, DCLK3, ABTB3, LRNN2, TMEM132E*, and *TAF5LP1*). Similarly, among the 200 stimulated genes (5–10‐fold change), 72 had no known link with IFN regulation (Table S1). Surprisingly, several canonical ISGs (*IFITM1, IFIT5, IFI16, IRF1, IRF2, IRF9, IRF2BPL*, and *STING1*) showed only modest change (<fivefold) suggesting cell specific regulation. Only two genes, *PNPT1* and *TYMP*, related to mitochondrial diseases, exhibited a 6.1‐ and 5.37‐fold change in IFN‐treated control fibroblasts.

### 2.2 Transcriptomic Analysis of Patients’ Fibroblasts

Fibroblasts from patients carrying biallelic pathogenic variants in *ATAD3A*, *DGUOK*, *MPV17*, *POLG*, and *PNPT1* (Table 1) displayed numerous deregulated genes compared with controls (Figure 1A). However, only a small subset corresponded to known ISGs (Figure 1A, cyan dots; Table S2), and GO, KEGG, and Reactome pathway analyses did not reveal strong enrichment of IFN signaling similar to that observed in IFN‐treated control fibroblasts (Figure 1B; Figure S1).

**TABLE 1:** List of cultured skin fibroblasts of patients and their genetic characterization.

Fibroblast name	Gender of patients	Gene	Gene nomenclature	Nucleotide change	Amino acid change
ATAD3A	M	ATAD3A	NM_018188.3	c.625_627del; c.251C>T	p.Glu209del; p.Thr84Met
DGUOK	F	DGUOK	NM_080916	c.708‐20A>G	
ELAC2‐1	M	ELAC2	M_018127.7	c.2249T>C	p.Met750Thr
ELAC2‐2	M	ELAC2	M_018127.7	c.591G>A; c.1943C>T	p.Trp197*; p.Ala648Val
ELAC2‐3	M	ELAC2	M_018127.7	c.1603G>T	p.Val535Phe
GTPBP3	F	GTPBP3	NM_133644.3	c.31_32insG	p.Gln11Argfs*98
MPV17	F	MPV17	NM_002437.5	c.498C>A	p.Asn166Lys
MRPL12	M	MRPL12	NM_002949.4	c.542C>T	p.Ala181Val
MTO1	F	MTO1	NM_001123226.1	c.1510C>T; c.1577C>T	p.Arg504Cys; p.Thr526Ile
MT‐TF	M	MT‐TF	NC_012920.1	m.616T>C	
NDUFA6	M	NDUFAF6	NM_152416.4	c.2.T>C; c.532G>C	p.Met1?; p.Ala178Pro
PNPT1	M	PNPT1	NM_033109.3	c.1160A>; c.1160A>G	p.Gln387Arg; p.Gln387Arg
PNPT1‐1	M	PNPT1	NM_033109.3	c.407G>A	p.Arg136His
PNPT1‐2	M	PNPT1	NM_033109.3	c.208T>C; c.2137G>T	p.Ser70Pro; p.Asp713Tyr
POLG‐1	M	POLG	NM_002693.1	c.1399G>A; c.695G>A	p.Ala467Thr; p.Arg232His
POLG‐2	M	POLG	NM_002693.1	c.2243G>C; c.3125dup	p.Trp748Ser; p.Val1043Glyfs*4
POLG‐3	M	POLG	NM_002693.1	c.428C>T; c.2542G>A	p.Ala143Val; p.Gly848Ser
POLG‐4	F	POLG	NM_002693.1	c.1682A>T; c.2243G>C	p.Lys561Met; p.Trp748Ser
POLG‐5	F	POLG	NM_002693.1	c.2554C>T; c.3286C>T	p.Arg852Cys; p.Arg1096Cys
POLG‐6	F	POLG	NM_002693.1	c.1399G>A; c.1283T>C	p.Ala467Thr; p.Leu428Pro
POLG‐7	F	POLG	NM_002693.1	c.1399G>A; c.3273+2T>C	p.Ala467Thr; splice
POLG‐8	F	POLG	NM_002693.1	c.1399G>A; c.975dup	p.Ala467Thr; p.Thr326Hisfs*62
POLG‐9	M	POLG	NM_002693.1	c.1399G>A; c.975dup	p.Ala467Thr; p.Thr326Hisfs*62
POLG‐10	F	POLG	NM_002693.1	c.803G>CC	p.Gly268Ala
POLG‐11	F	POLG	NM_002693.1	c.3314C>T; c.1760C>T	p.Ala1105Val; p.Pro587Leu
POLG‐12	F	POLG	NM_002693.1	c.2420G>C; c.3550G>A	p.Arg807Pro; p.Asp1184Asn
TFAM	M	TFAM	NM_003201.2	c.458G>A	p.Gly153Glu
TOP3A	F	TOP3A	NM_004618.4	c.408G>T; c.1982G>A	p.Glu136Asp; p.Cys661Tyr
TRMT10C	F	TRMT10C	NM_017819.3	c.542G>T	p.Arg181Leu
TRMU	M	TRMU	NM_018006.5	c.2T>A	p.Met1?
TWNK	M	TWNK	NM_021830.5	c.1370C T	p.Thr457Ile

*Note*: The PNPT1 cell line was used only for RNAseq analysis.

### 2.3 IFN‐Related Biomarkers in Patients’ Fibroblasts

**FIGURE 2:**
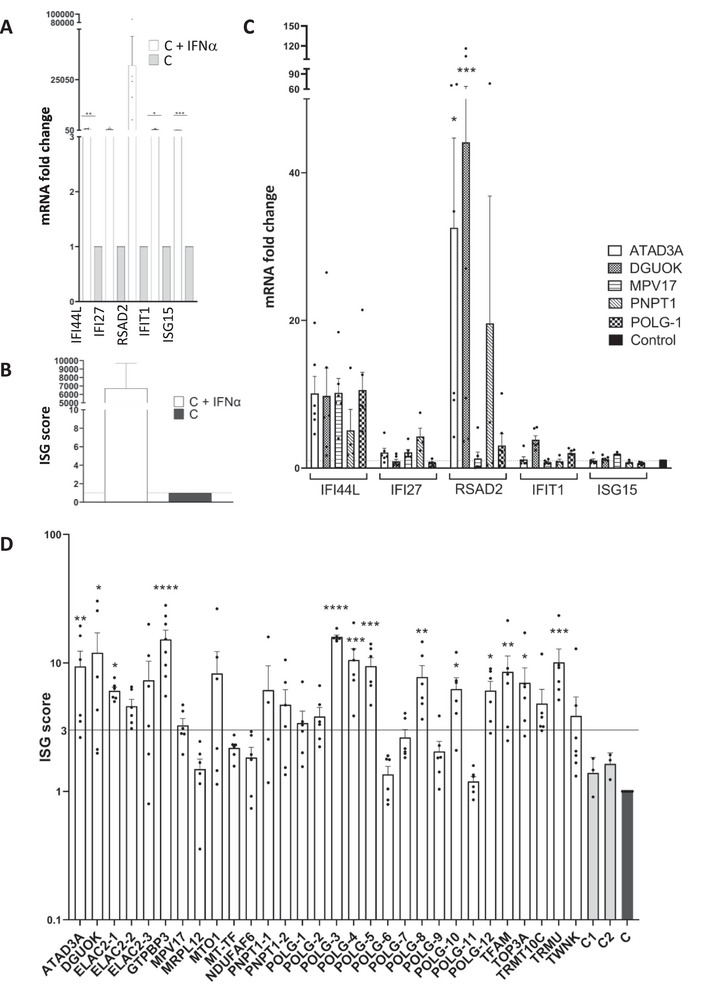
IFN signaling in controls and patients’ fibroblasts. **(A)** RT‐qPCR analysis of 5 ISGs in IFN‐treated vs. nontreated control fibroblasts normalized to HPRT mRNA. Mean values of five successive passages are shown. Statistical significance (*p < 0.05, **p < 0.01, ***p < 0.001) in paired *t*‐test. **(B)** ISG score (mean fold change of mRNA levels of the five ISGs) in IFN‐treated vs. nontreated control fibroblasts. Mean values of five successive passages are shown. Paired *t*‐test was used for statistical analysis (*p*‐value 0.086). The control value has been arbitrarily set to 1. **(C)** RT‐qPCR analysis of five ISGs in fibroblasts of five patients normalized to HPRT mRNA. Mean values of 4–6 successive passages are shown. Statistical significance (*p < 0.05, **p < 0.01, ***p < 0.001) in one‐way ANOVA. **(D)** ISG score in various mutant fibroblasts. The mean value of one control has been arbitrarily set to 1, and two additional controls are also shown. Mean values and data points of 4–8 successive passages are shown. Statistical significance (*p < 0.05, **p < 0.01, ***p < 0.001) in the Kruskal–Wallis test compared with the control. The horizontal line indicates an ISG score of 3.

To screen IFN activation more directly, we quantified five established ISG biomarkers (*IFI27*, *IFI44L*, *IFIT1*, *ISG15*, and *RSAD2*) by quantitative RT‐PCR (Q‐RT‐PCR) [19, 20]. IFN‐treated control fibroblasts showed marked induction, validating the model (Figure 2A). The ISG score, calculated as the mean fold change of the biomarkers compared with nontreated controls, reached 6000 in IFN‐treated fibroblasts (Figure 2B) and was also elevated in mycoplasma‐contaminated cells (Figure S2). An ISG score >3 was considered increased, based on scores of mutant ATAD3A (9.39) and PNPT1 cells (6.14 and 4.13) known to present increased IFN response [2, 3]. Studying the ISG score of the patient fibroblasts, we found an increased expression of *IFI44L* in 4/5 cell lines and variable levels of *IFI27*, *RSAD2, IFIT1*, and *ISG15* expression (Figure 2C). Among 30 patient fibroblasts carrying defects of mitochondrial translation (*MT‐TF*, *MRPL12*), mtDNA maintenance (*ATAD3A*, *DGUOK, MPV17*, *POLG, TFAM*, *TOP3A*, and *TWNK*), mtRNA processing (*ELAC2*, *GTPBP3*, *MTO1*, *PNPT1*, *TRMT10C*, and *TRMU*), and respiratory chain assembly (*NDUFAF6*), 43% exhibited significantly elevated ISG scores, particularly those with variants in *ATAD3*, *DGUOK*, *ELAC2*, *GTPBP3*, *TFAM*, *TOP3A*, and *TRMU* (Figure 2D). However, IFN activation was inconsistent in POLG fibroblasts, especially for two siblings with identical POLG variants (POLG‐8 and POLG‐9 have increased and normal ISG scores, respectively). TBK1 phosphorylation analysis did not correlate clearly with IFN upregulation (Figure S3).

### 2.4 Relationship Between ISG Score and mtDNA Leakage

**FIGURE 3:**
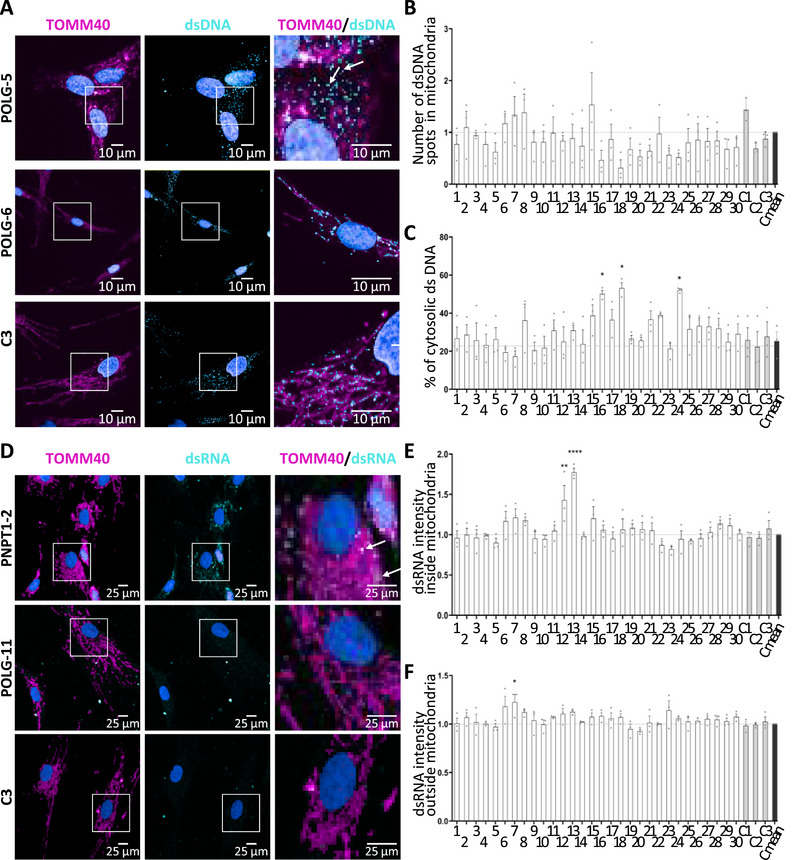
**Cytosolic release of mitochondrial dsDNA and dsRNA. (A)** Representative images of immunofluorescence staining of mitochondria (TOMM40), dsDNA, and nucleus (DAPI) in POLG‐5, POLG‐6, and control fibroblasts observed by confocal microscopy. Arrows indicate the cytosolic dsDNA spots, original magnification 60×, scale bar: 10 µm. **(B)** Quantification of mitochondrial dsDNA spots using the Signals Image Artist software (Revvity, v1.4.2) and **(C)** percentage of dsDNA in the cytosol relative to total spots. **(D)** Representative images of immunofluorescence staining of mitochondria (TOMM40), dsRNA, and nucleus (DAPI) in PNPT1‐2, POLG‐11, and control fibroblasts. Arrows indicate the mitochondrial dsRNA spots, original magnification 20×, scale bar: 25 µm. Quantification of dsRNA inside **(E)** and outside mitochondria **(F)** using the Signals Image Artist software (Revvity, v1.4.2). The total dsRNA intensity in the various patients’ fibroblasts is normalized to control cells. Results averaged from at least 7 cells counted from one experiment, representative of three independent experiments. Statistical significance (****p < 0.0001) in one‐way ANOVA with Dunnett's multiple comparison test. 1: ATAD3A, 2: DGUOK, 3: ELAC2‐1, 4: ELAC2‐2, 5: ELAC2‐3, 6: GTPBP3, 7: MPV17, 8: MRPL12, 9: MTO1, 10: MT‐TF, 11: NDUFA6, 12: PNPT1‐1, 13: PNPT1‐2, 14: POLG‐1, 15: POLG‐2, 16: POLG‐3, 17: POLG‐4, 18: POLG‐5, 19: POLG‐6, 20: POLG‐7, 21: POLG‐8, 22: POLG‐9, 23: POLG‐10, 24: POLG‐11, 25: POLG‐12, 26: TFAM, 27: TOP3A, 28: TRMT10C, 29: TRMU, 30: TWNK.

**FIGURE 4:**
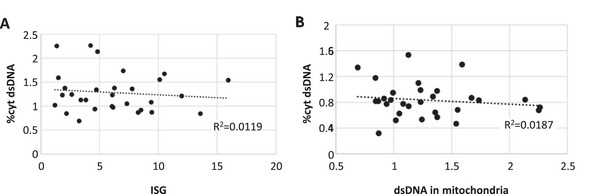
Correlation analysis between ISG score and cytosolic dsDNA content (**A**) or mitochondrial and cytosolic dsDNA content (**B**). *R* ^2^ values were calculated using Excel.

Because cytosolic mtDNA can trigger an IFN response, we examined mtDNA and mtRNA localization in patient cells by confocal microscopy. Significant increase of cytosolic double‐strand DNA (dsDNA) was found in POLG‐3, POLG‐5, and POLG‐11 fibroblasts (Figure 3A,C). MRPL12, POLG‐2, POLG‐4, POLG‐8, and POLG‐9 showed a trend toward cytosolic mtDNA leakage (Figure 3C). The other cell lines showed no evidence of cytosolic mtDNA leakage, but no consistent correlation was observed between ISG scores and mtDNA leakage (Figure 4A). We also observed a significant decrease in dsDNA in mitochondria of POLG‐5 fibroblasts (Figure 3B), but no correlation could be established between cytosolic mtDNA leakage and total mtDNA content (Figure 4B). Moreover, several fibroblasts demonstrated a slight accumulation of mitochondrial dsRNA, especially the two PNPT1 mutant cell lines (Figure 3D,E), as previously reported [2]. Extramitochondrial mtdsRNA was detected in PNPT1 mutant fibroblasts and in POLG‐10 cells, but was not significantly different from control, contrary to MPV17 fibroblasts, characterized by a normal ISG score (Figure 3D,F). Therefore, cytosolic mtRNA leakage represents a rare event and is not correlated to upregulation of ISGs.

## 3 Discussion

Here, we investigate human cultured skin fibroblasts as a potential biological resource for the investigation of IFN signaling. We show that exogenous type I IFN elicited a more than >10‐fold change in both known and previously unreported inducible genes in control cultured skin fibroblasts. A spontaneous and significant type I IFN signature similar to that observed in fibroblasts of patients with type I interferonopathy (*LSM11*, *RNU7‐1* [21], and *RNASEH2* [22]) was found in 43% of fibroblasts from patients with pathogenic variants in the *ATAD3A*, *DGUOK, ELAC2, GTPBP3, POLG, TFAM, TOP3A*, and *TRMU* genes. Increased type I IFN response has been previously reported in ATAD3A cultured fibroblasts [3], in the muscle of patients with Chronic Progressive External Ophthalmoplegia, MELAS syndrome [13], and in whole blood samples of several patients with mitochondrial diseases of various genetic etiologies [16].

The present study only gives partial support to the relevance of primary cultured skin fibroblasts for investigating IFN response in patients’ cell lines, as increased ISG score was not a consistent feature in all patients. Similarly, discrepant results were observed in two POLG sibs sharing the same genotype and a similar clinical presentation [23]. Along those lines, fibroblasts carrying compound heterozygote pathogenic *POLG* variants (p.Trp748Ser and a nonsense variation p.Val1043Glyfs*4 in our POLG‐2 cell line) reportedly showed an IFN response similar to controls, as previously reported in MIRAS patients homozygous for the p.Trp748Ser pathogenic variant [24]. On a different genetic background, the Trp748Ser variant resulted in a high‐ISG score in combination with the p.Lys561Met variant in our patient POLG‐4 cell lines (ISG:10.5). Why identical pathogenic variants on distinct genetic backgrounds cause discrepant IFN responses may be possibly accounted for by hitherto unexplained epistatic mechanisms.

None of the patients with a high ISG score in fibroblasts has clinical or biological symptoms suggestive of inflammation and/or autoimmune disease, questioning the actual impact of increased type I IFN signaling in the context of mitochondrial diseases. Yet, overlapping features, especially neuroimaging findings [25] observed in both mitochondrial diseases and type I interferonopathies, suggest that an elevation of CSF IFN may have an infraclinical, possibly actionable impact. Conversely, one previously reported POLG patient (POLG‐1) with polyradiculoneuritis [23, 26] and brain MRI evidence of neuroinflammation failed to display an elevated ISG score in his cultured fibroblasts. Moreover, while pathogenic POLG variants were associated with distinct clinical courses dominated by neurological, hepatic, and gastrointestinal symptoms in our series [23], we failed to make any correlation between clinical course and ISG scores in cultured cell lines of our patients.

In autoinflammatory diseases such as type I interferonopathies, AGS, and lupus, where upregulated IFN signaling is posited as relevant to pathogenesis, the IFN status is determined by measuring the ISG score in blood. Most of the patients presented here passed away early, hampering specialized in vivo analyses. Future combined measurement of ISG scores in blood and cultured fibroblasts should help decide which sample is best suited for screening of IFN response and whether its induction is gene or genotype‐specific in mitochondrial disease, as discussed in AGS patients carrying *LSM11* and *RNU7‐1* pathogenic variants [21], and ATAD3A [3] or PNPT1 patients [2].

Leakage of mtDNA or mtRNA in the cytosol is known to induce an IFN response. In our study, no clear correlation between cytosolic mtDNA leakage and IFN response could be established. Although some fibroblasts with mtDNA leakage also have increased ISG (POLG‐3, POLG‐4, POLG‐5, and POLG‐8), others have high ISG but no mtDNA leakage (ATAD3A, DGUOK), or normal ISG but mtDNA leakage (MRPL12 and POLG‐11). At variance with the high increase of cytosolic mtDNA reported in *ATAD3A*‐silenced human fibroblasts [3] or in heterozygote deletions of the *Tfam* gene in MEF cell lines [1], we were unable to detect mtDNA leakage in ATAD3A or TFAM patients’ cell lines.

In parallel, we observed cytosolic mtRNA leakage only in MPV17 fibroblasts. Finally, while downregulation of *PNPT1* resulted in a fourfold cytosolic increase of dsmtRNA in HeLa cells and patients’ fibroblasts [2], we did observe a similar, but not significant, increase of mtRNA. Nevertheless, the two PNPT1 cell lines displayed an important accumulation of intramitochondrial dsRNA. Our results suggest that in patients’ cell lines, biallelic pathogenic variants altered IFN regulation in a different way than gene silencing in control fibroblasts. It is also possible that some hitherto unknown tissue‐specific factors account for those discrepancies. If confirmed by others, these results would question the relevance of cellular models as illustrators of pathological situations in humans.

In conclusion, we show here that IFN response was increased in cell lines of several but not all patients carrying genetically identified mitochondrial defects. Cytosolic accumulation of mtDNA and mtRNA was inconsistently observed and largely unrelated to ISG scores. Our findings support the view that altered IFN signaling in cultured cell lines is a yet inconstant feature in mitochondrial diseases. Future studies will hopefully shed light on the clinical impact and relevance of impaired IFN signaling and which cultured cell type is best suited for the study of mitochondrial diseases.

## 4 Material and Methods

### 4.1 Fibroblast Cell Lines

Table 1 shows the genotypes of 31 primary cultured skin fibroblast cell lines derived from patients carrying biallelic pathogenic variants in mitochondrial disease‐causing genes. M: male, F: female. The cell lines were free of mycoplasma contamination.

### 4.2 Cell Culture and Treatments

Primary cultured skin fibroblasts were grown as previously described [27]. Control cells were stimulated with IFNα (IntronA) 10,000 U/mL for 6 h. Phosphorylated TBK1 analysis was performed on cells supplemented with 500 ng/mL diABZI for 2 h.

Forward (YGCCTGVGTAGTAYRYWCGC) and reverse primers (GCGGTGTGTACAARMCCCGA) were used for the detection of various mycoplasma strains.

### 4.3 Protein Extraction and Immunoblotting

Cells were lysed in RIPA buffer supplemented with Halt Protease and Phosphatase inhibitor cocktails [1X], 2 [1X], and 3 [1X] were added to the cell pellet for 20 min on ice, then centrifuged at 20,000*g* for 5 min at 4°C. Lysed cells were sonicated (30 s on and 30 s off, 10 times). The samples were loaded on 4%–15% gel (Bio‐Rad) and transferred to a nitrocellulose membrane. The membranes were saturated with 5% BSA in TBS+0.1% Tween. Antibodies were rabbit anti‐TBK1 (ab40676, Abcam, 1:1000 dilution), rabbit anti‐P‐TBK1 (#5483, Cell Signaling, 1:1000 dilution), and mouse anti‐GAPDH (ab8245, Abcam, 1:500 dilution) and incubated overnight at 4°C. The secondary antibodies were anti‐rabbit IgG HRP and anti‐mouse IgG HRP (1:5000 dilution). Signal was detected using the Fusion FX machine using HRP detection with Clarity Max ECL (Bio‐Rad).

### 4.4 RNA Extraction

Total RNAs from cultured skin fibroblasts were extracted using a RNeasy mini kit (Qiagen) and treated with DNAse (Qiagen) according to the manufacturer's protocol. A same amount of RNA (1 µg) was used for reverse transcription.

### 4.5 Transcriptomic Analysis

RNA‐seq library preparation and sequencing were performed as previously described using 800 ng RNA [28]. RNA‐seq alignment and bioinformatics data analyses were done as previously described [29].

### 4.6 ISG Score

cDNA was synthesized using QuantiTect Reverse Transcription Kit (205313, Qiagen) using 1 µg RNA. Quantitative real‐time PCR (qPCR) was performed using Master Mix TaqMan (4440048, Applied Biosystems). The ISG score was determined by analyzing *IFI44L*, *IFI27*, *RSAD2*, *IFIT1*, and *ISG15* gene expression (TaqMan probes from Thermo Fisher Scientific; Table S3). The relative abundance was normalized to the internal control *HPRT1* (ΔC*t*), and mRNA expression levels in mutant cells were expressed relative to control (ΔΔC*t*). The fold change was then calculated with the formula 2^−ΔΔC^ *^t^*. The qPCR was done on the Viia7 Real Time machine (Thermo Fisher Scientific), and at least >6 determinations on successive passages were performed.

### 4.7 RNA and DNA Immunofluorescence Staining

Fibroblasts were grown in an Opera 96‐well plate, washed, and fixed with 4% paraformaldehyde (sc‐281692, ChemCruz) for 15 min. After three washes with PBS+1% BSA, cells were permeabilized with 0.1% Triton X‐100 in PBS+1% BSA for 10 min, washed, and incubated overnight at 4°C with primary antibody mouse monoclonal anti‐dsDNA (CBL186, Merck) or mouse monoclonal anti‐dsRNA J2 (10010200‐200UG, ThermoFischer) and rabbit polyclonal anti‐TOMM40 (18409‐1‐AP, Proteintech), 1:1000 dilution. Specificity of anti‐dsDNA and anti‐dsRNA antibodies was tested in human fibroblasts (Figure S4). After three washes with PBS+1%BSA, cells were incubated for 45 min with secondary antibody: goat anti‐Mouse IgM, Alexa Fluor 594 (1:1000 dilution), and goat anti‐mouse Alexa Fluor 488, goat anti‐rabbit Alexa Fluor 488, and goat anti‐rabbit Alexa Fluor 568 (1:500 dilution). Cells were then incubated with WGA 647 (W32466, Thermo Fisher, 45 min, dilution 1:200) for cytoplasm staining and DAPI (5 min at 1:1000) for nucleus staining.

### 4.8 High‐Content Imaging and Analysis

For quantification, plates were imaged with an Opera Phenix Plus (Revvity) running in Harmony v5.23, in confocal (spinning‐disk) mode, with a 20×/NA 1 or 63×/1.15 water immersion objectives (for RNA or DNA imaging, respectively). Fluorescent channels were chosen as follows: DAPI (Excitation/Emission filter wavelengths (Exc/Em), in nm): 405/435‐480; Alexa 488: 488/500‐550; Alexa 594: 565/570‐630; Alexa 647: 633/650‐760. A small Z‐stack of three planes was acquired. Forty‐two fields of view (FoV)/Z‐stack with 0.5 µm step or nine FoV/Z‐stack with 0.8 µm step were acquired per well, for DNA or RNA imaging, respectively.

All images were transferred and analyzed using the Signals Image Artist software (Revvity, v1.4.2). From the maximal intensity projection images, nuclei were segmented, filtered, and the corresponding cytoplasm was then recognized from them. Only complete cells, not touching the image borders, were further processed. Mitochondria were then segmented to delimit subcellular regions, and various morphological and intensity parameters were calculated. For DNA analysis, an additional step was performed to recognize DNA “spots” and categorize them based on their location (inside or outside mitochondria).

### 4.9 Statistics

Student's *t*‐tests, ANOVA, and Kruskal–Wallis tests were performed with GraphPad Prism (version 9.5.1) software. Data presented are based on three to eight independent experiments (mean ± SEM).

## Author Contributions

Manon Marchais and Alexandre Pierga: Investigation, methodology. Alessandra Pennisi: Investigation. Alice Lepelley: Writing – review and editing. Nicolas Cagnard, Christine Bole, and Patrick Nitschke: Data curation. Mohamed Hamici: Data curation. Frédéric Rieux‐Laucat: Conceptualization, funding acquisition, writing – review and editing. Manuel Schiff and Arnold Munnich: Writing – review and editing. Agnès Rötig: Conceptualization, supervision, funding acquisition, writing – original draft.

## Funding

We thank Emilie Ouanounou for helping with the cell culture. This study was supported by the Imagine Institute, the Agence Nationale de la Recherche through the Investissements d'Avenir program ANR‐10‐IAHU‐01, and the E‐Rare project GENOMIT (01GM1207). The funders of the study had no role in study design, data collection, data analysis, data interpretation, or writing of the report. The corresponding author had full access to all the data in the study and had final responsibility for the decision to submit for publication.

## Ethics Statement

The study complied with the Declaration of Helsinki and was approved by the local Institutional Review Boards of each hospital.

## Patients’ Consent

Written informed consent was obtained from parents before genetic testing.

## Conflicts of Interest

The authors declare no conflicts of interest.

## Supporting information




**Supporting File**: eji70176‐sup‐0001‐SupMat.pdf.

## Data Availability

The data underlying this article will be shared on reasonable request to the corresponding author. The RNAseq data generated in this study have been deposited at BioStudies (accession number: S‐BSST2256).
