# Supplementary material for: No Correlation Between Interferon Signaling and Cytosolic Mitochondrial DNA/RNA Leakage in Cultured Skin Fibroblasts of Patients With Mitochondrial Diseases
Source: Eur J Immunol. 2026 Mar 27;56(4):e70176. doi: 10.1002/eji.70176 (PMC13022800; doi:10.1002/eji.70176)
Supplement: Supplementary file 1 — Supporting File: eji70176‐sup‐0001‐SupMat.pdf. [file EJI-56-e70176-s001.pdf]

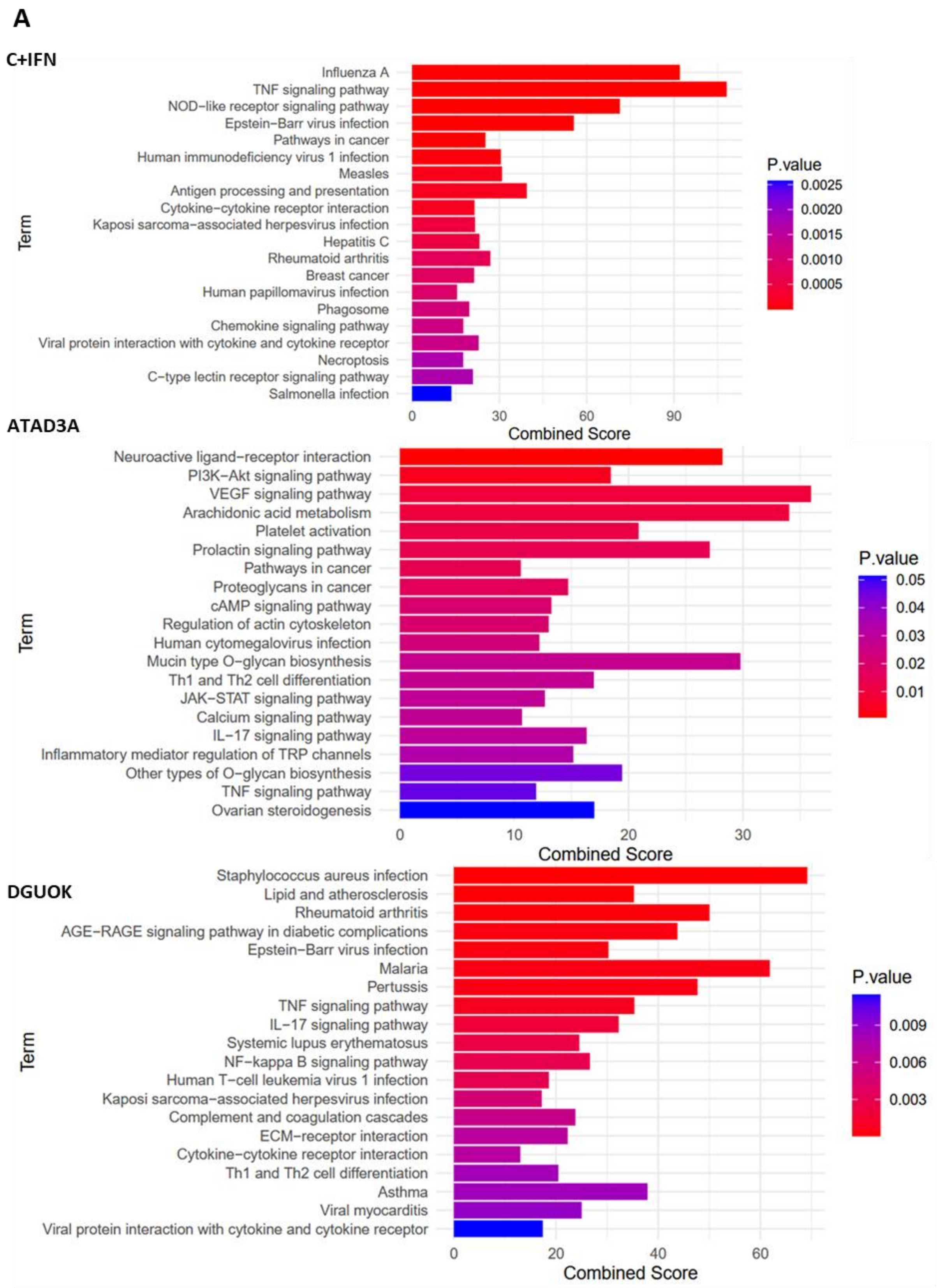

S1 Fig. Functional enrichment analysis of DEGs in IFN-treated control fibroblasts (C+IFN), ATAD3A, DGUOK, MPV17, PNPT1 and POLG fibroblasts vs control fibroblasts by KEGG (**A**) and Reactome (**B**).

A (follow)

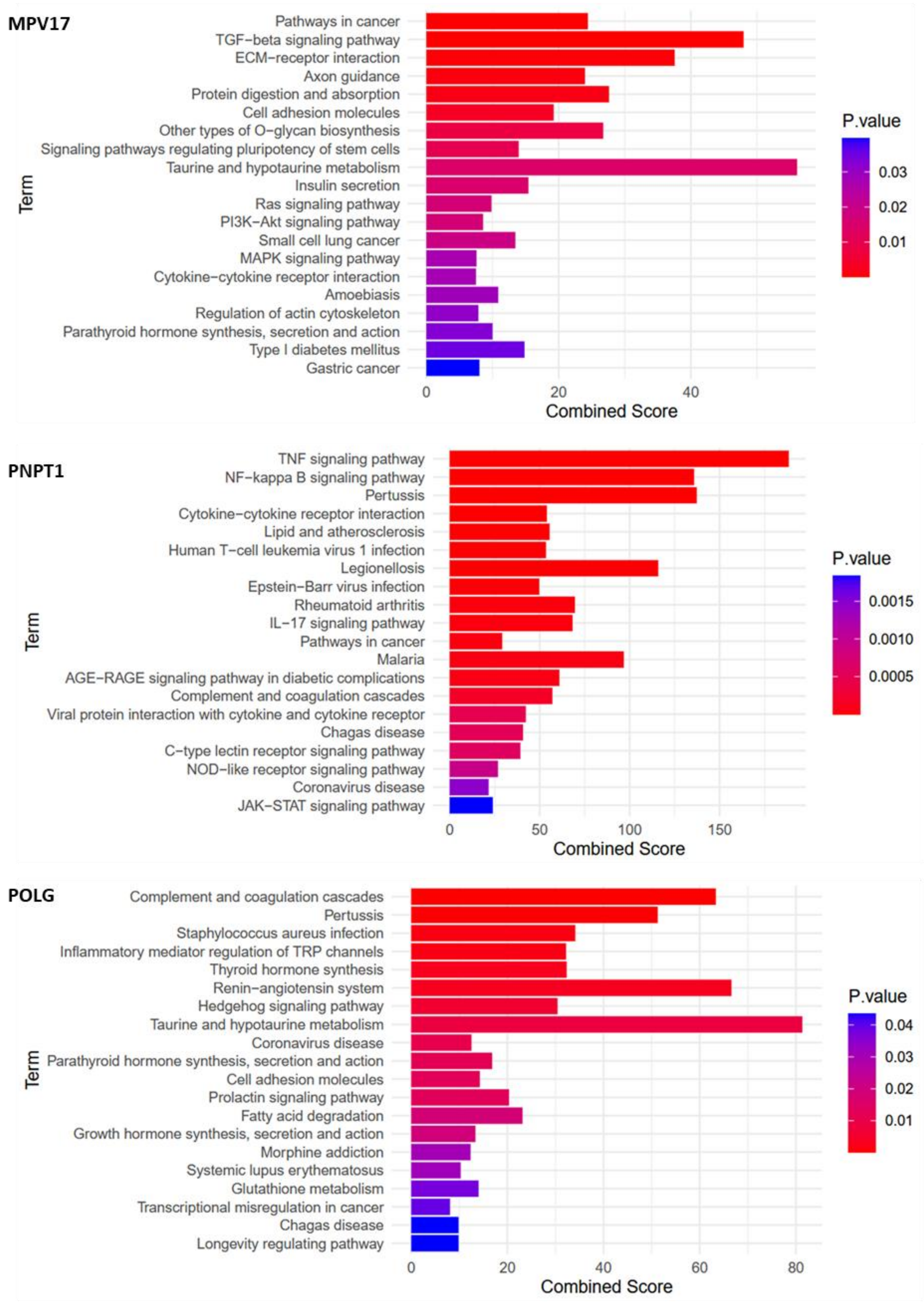

S1 Fig. Functional enrichment analysis of DEGs in IFN-treated control fibroblasts (C+IFN), ATAD3A, DGUOK, MPV17, PNPT1 and POLG fibroblasts vs control fibroblasts by KEGG (**A**) and Reactome (**B**).

B

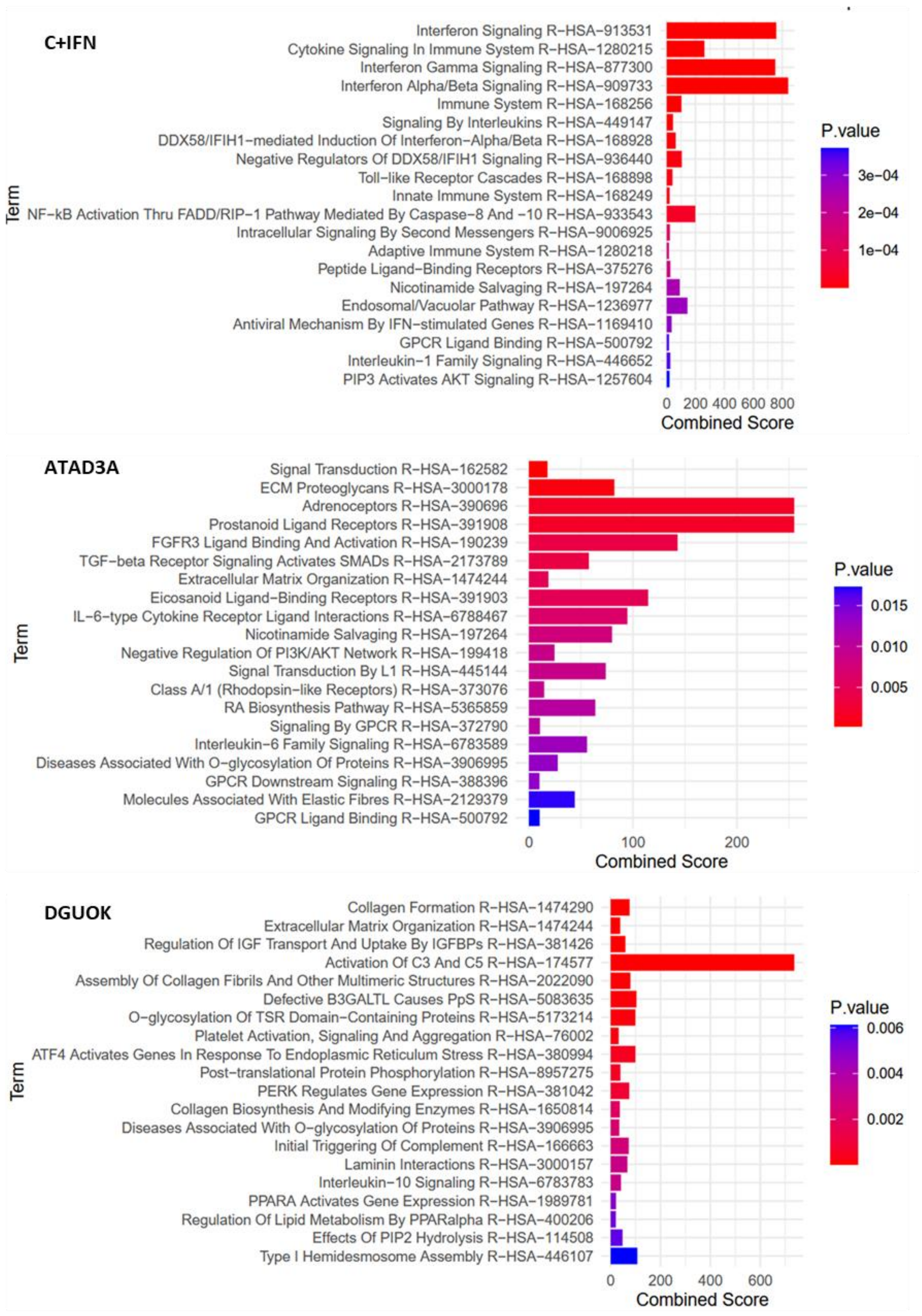

S1 Fig. Functional enrichment analysis of DEGs in IFN-treated control fibroblasts (C+IFN), ATAD3A, DGUOK, MPV17, PNPT1 and POLG fibroblasts vs control fibroblasts by KEGG (**A**) and Reactome (**B**).

B (follow)

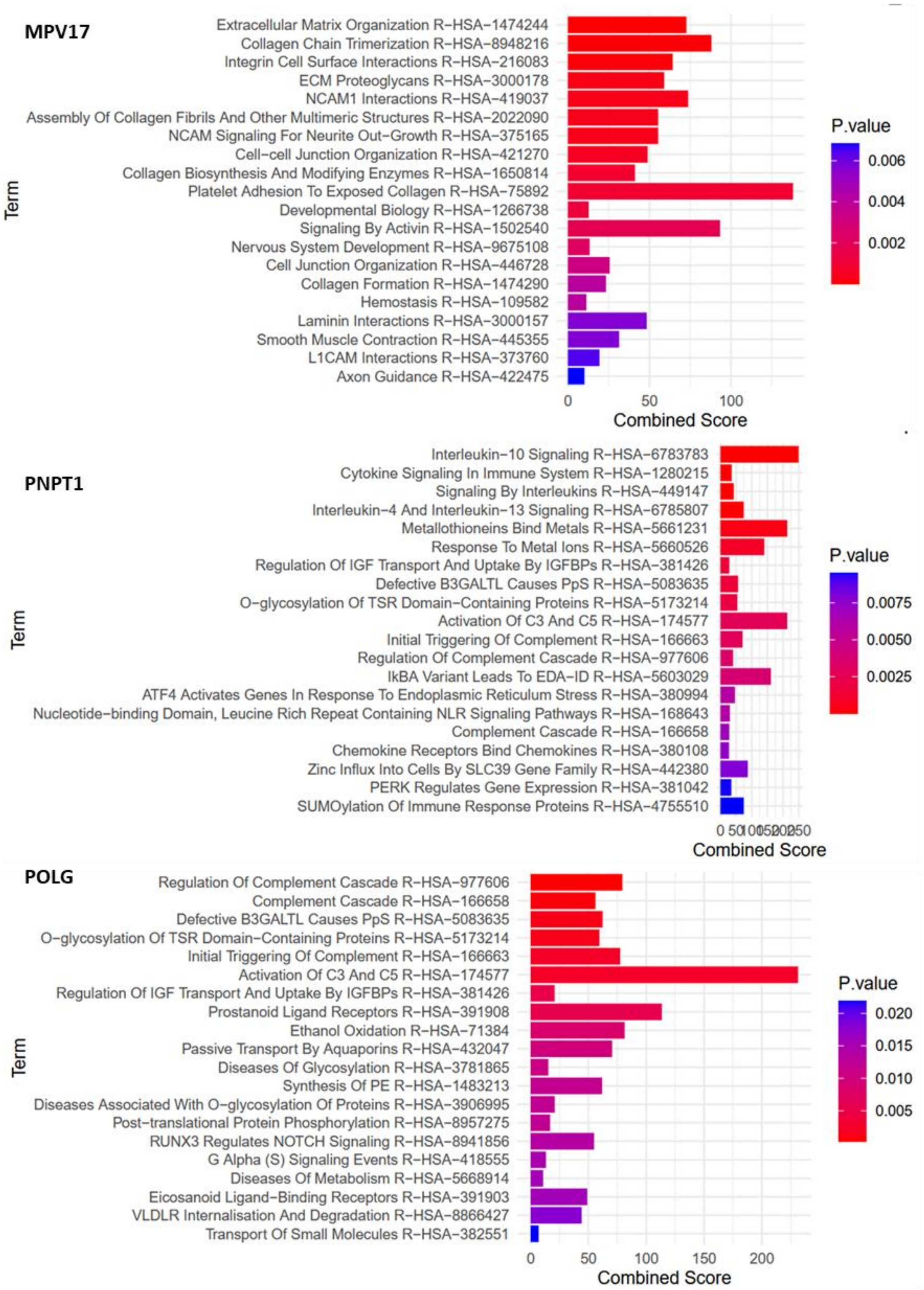

S1 Fig. Functional enrichment analysis of DEGs in IFN-treated control fibroblasts (C+IFN), ATAD3A, DGUOK, MPV17, PNPT1 and POLG fibroblasts vs control fibroblasts by KEGG (**A**) and Reactome (**B**).

A

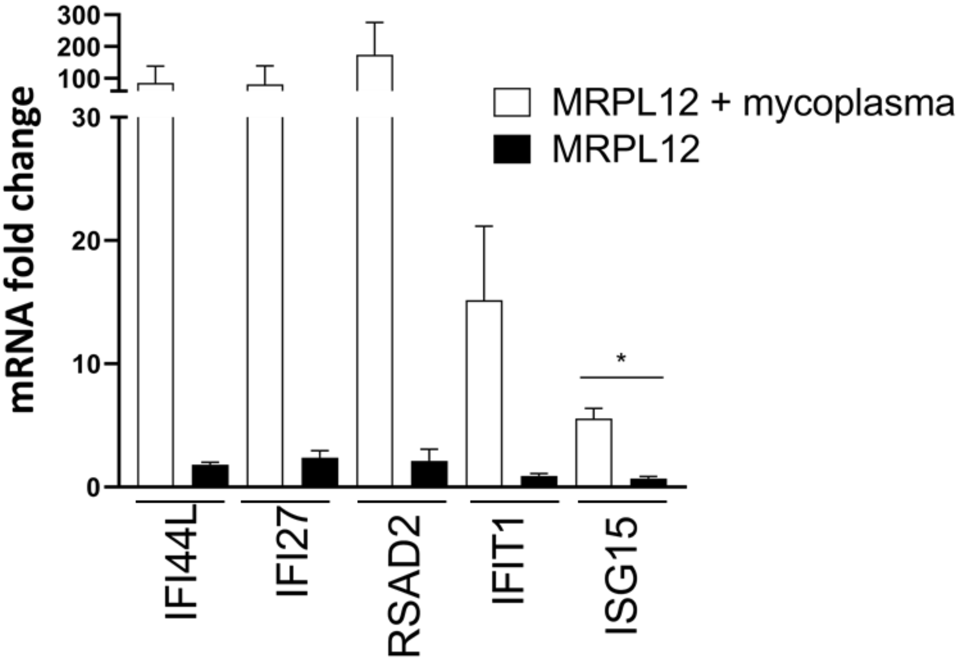

B

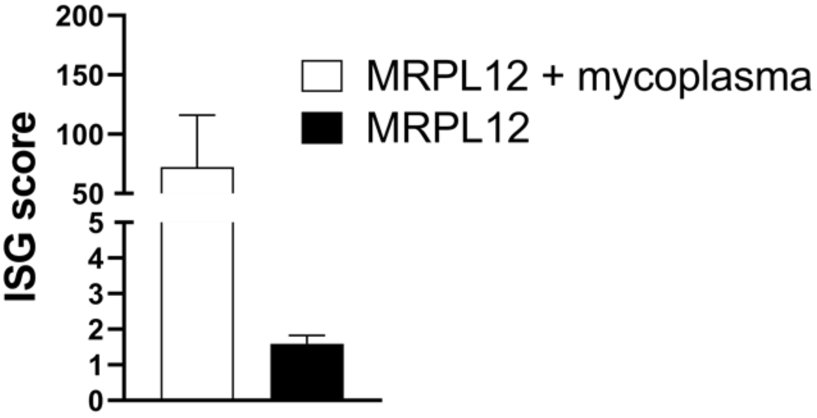

S2 Fig. IFN signaling in mycoplasma contaminated fibroblasts. (A) Expression of IFI44L, IFI27, RSAD2, IFIT1 and ISG15. (B) ISG score (i.e. the median fold change of mRNA levels of the five ISGs in MRPL12 fibroblasts contaminated (MRPL12 +mycoplasma) or not by mycoplasma (MRPL12). * indicates statistical significance ($P<0.05$) in paired t-test.

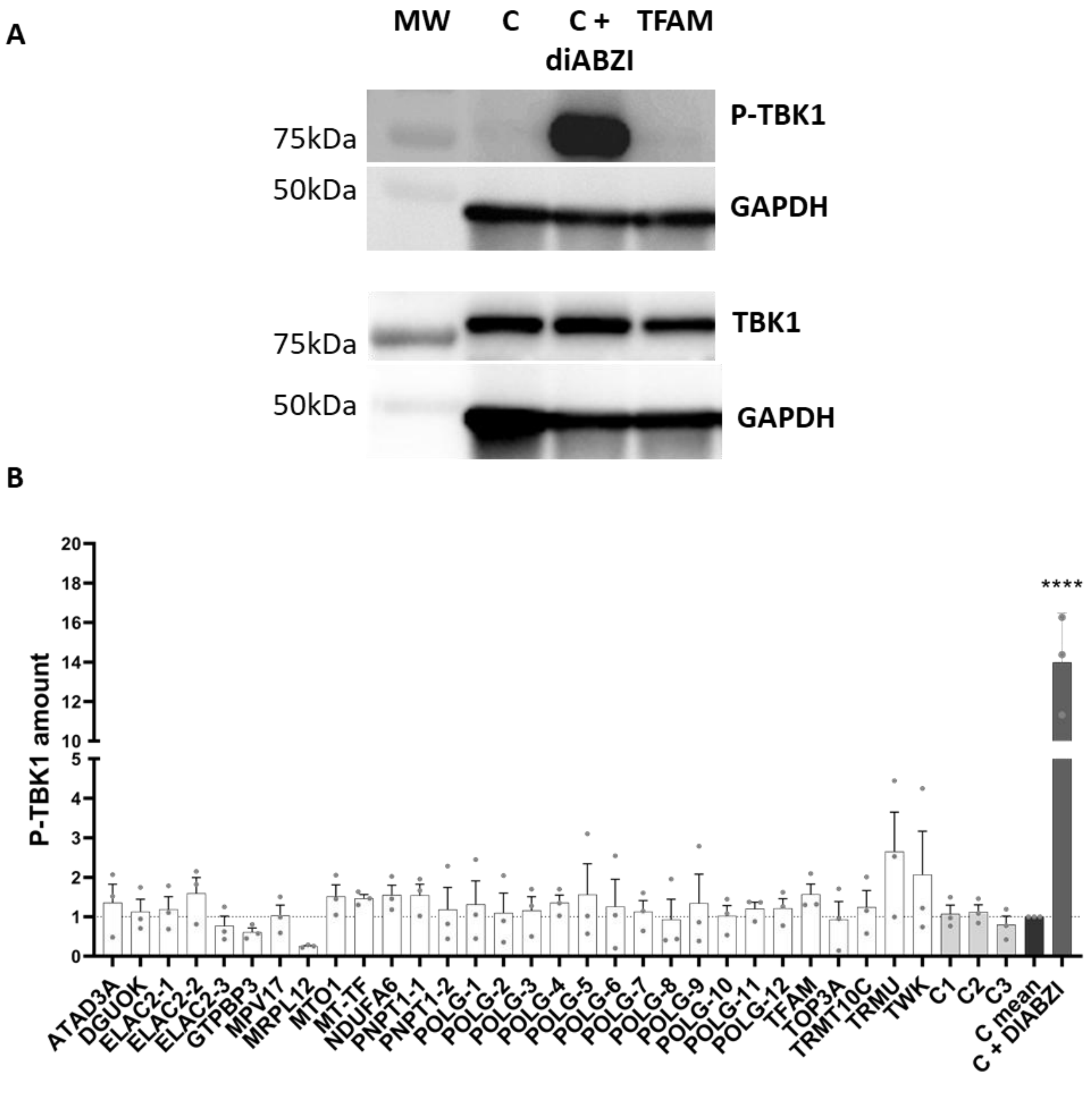

S3 Fig Analysis of phosphorylated TBK1. (A) Representative analysis by western blot of TBK1 and phosphorylated TBK1 (P-TBK1) in control, control + diABZI, PNPT1-1, ELAC2-1 and ELAC2-2 fibroblasts. GAPDH was used as loading control. Blots are representative of three independent experiments. Lanes were cropped to show only the specific signal detected by each antibody. **(B)** Quantification of phosphorylated TBK1 in patients' and controls fibroblasts treated or not with diABZI. The mean value of one control has been arbitrarily set to 1 and three additional controls are also shown. P-TBK1 level was normalized to TBK1 level. Blots are representative of three independent experiments. **** indicate statistical significance (P<0.0001) in one way ANOVA.

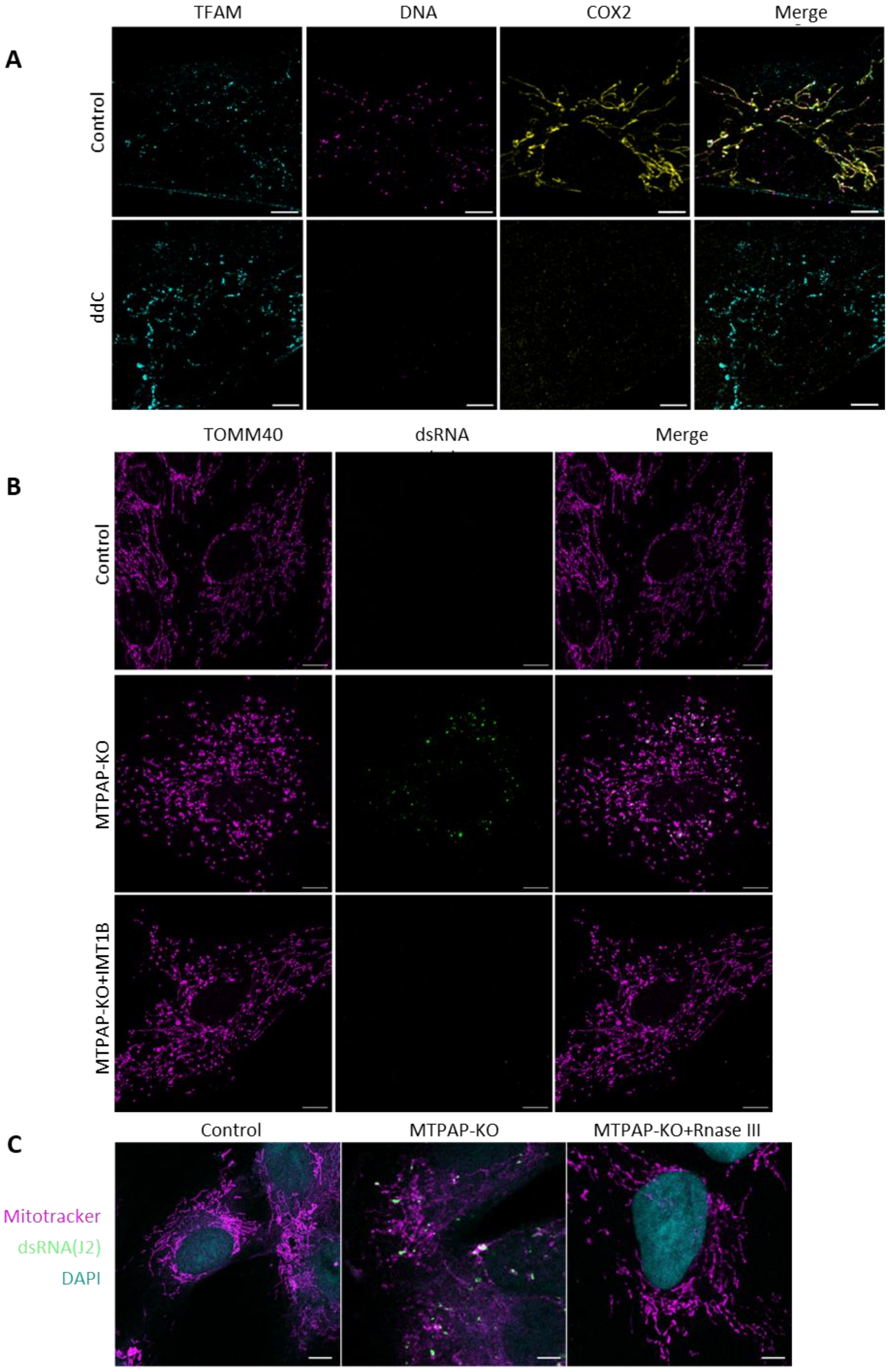
A
TFAM
DNA
COX2
Merge
Control
ddC
B
TOMM40
dsRNA
Merge
Control
MTPAP-KO
MTPAP-KO+IMT1B
C
Control
MTPAP-KO
MTPAP-KO+Rnase III
Mitotracker
dsRNA(J2)
DAPI

S4 Fig. Specificity of anti-dsDNA and anti-dsRNA antbodies.

A. Representative images of healthy donor fibroblasts stained with anti-TFAM antibody (C9 clone, Santa Cruz, sc-376672), anti-DNA antibody (AC-30-10, Merck, CBL186) and anti-COX2 antibody (Proteintech, 55070-1-AP). Top panel: cells were kept in culture at 37°C, 5% CO_2 in DMEM, 10%SVF, 50 µg/mL uridine, 1 mM sodium pyruvate. Bottom panel: cells were kept in the same medium supplemented with 100 µM dideoxycytidine (ddC, Sigma-Aldrich) for 10 days to deplete mtDNA. Note the complete depletion of mtDNA and mitochondrially encoded COX2 in the ddC treated condition while nuclear encoded TFAM is still present. Scale bar: 5 µm.

B. Representative images of BJ-5ta human fibroblasts stained with anti-TOMM40 antibody (Proteintech, 18409-1-Ap), anti-dsRNA antibody (J2, Jena Bioscience, RNT-SCI-10010200). Top panel is control BJ-5ta, in middle panel MTPAP has been knocked out by CRISPR (note the accumulation of dsRNA in the mitochondria in this condition), bottom panel is MTPAP-KO cells treated with POLRMT inhibitor IMT1B for 48 h preventing mitochondrial transcription (Medchem, HY-137067), note the depletion of dsRNA in this condition. Scale bar: 5 µm.

C. Representative images of BJ-5ta human fibroblasts stained with Mitotracker (ThermoFisher, M22426), anti-dsRNA antibody (J2, Jena Bioscience, RNT-SCI-10010200) and DAPI (ThermoFisher, D1306). Left panel is control BJ-5ta, in middle panel MTPAP has been knocked out by CRISPR (note the accumulation of dsRNA in the mitochondria in this conditions), right panel is MTPAP-KO cells treated post fixation and permeabilization with dsRNA specific ribonuclease RNAse III (NEB, M0245S), note the depletion of dsRNA in this condition. Scale bar: 5 µm.

Supplementary Table 1. Genes with >5-fold-change up-regulation (logFC>2.3, p-value<0.05) in IFN-treated control fibroblasts.
* and x indicate genes present in the interferome database(1) or reported to be ISGs(2). Genes in bold have never been reported as IFN regulated or regulator genes.

Symbol		Reference	LogFC	p_value
OR52K3P		(3)	6.49	5.43E-05
CXCL11	*x		5.57	4.22E-03
CX3CL1	*x		5.54	1.37E-02
GBP6	*		5.40	8.78E-04
CXCL9	*x		5.11	8.76E-07
LIPG	*		5.09	5.50E-03
XIRP1	*		4.98	1.18E-02
CXCL10	*x		4.89	1.37E-02
**LINC01629**			4.70	9.80E-03
PAX5		(4)	4.63	2.39E-05
SSTR2		(5)	4.55	1.06E-02
KIF5C	*		4.34	3.25E-03
AQP3		(6)	4.21	2.60E-07
IDO1	*x		4.21	1.68E-02
NFATC2		(7)	4.11	1.12E-07
PIK3AP1		(8)	4.08	4.18E-09
GBP1P1	*		4.05	2.39E-06
GBP1P1	*		3.98	4.08E-08
IL33	*		3.96	1.29E-02
HERC6	*x		3.89	1.41E-02
TNFSF10	*x		3.84	2.90E-09
**DCLK3**			3.81	1.03E-06
CCL7	*		3.77	1.42E-02
SLFN12L		(9)	3.76	8.39E-07
LGALS9	*x		3.75	4.99E-11
MX2	*x		3.75	1.48E-02
HAS1		(10)	3.75	1.49E-06
GBP4	*x		3.73	8.05E-06
PHOSPHO1		(11)	3.70	5.79E-04
TNFSF13B	*x		3.68	4.20E-03
ARL9	*		3.63	6.68E-08
KLF14		(12)	3.60	4.09E-03
IL1RL1	*		3.60	9.24E-08
**ABTB3**			3.59	1.65E-05
GRIP2	*		3.59	7.20E-09

BISPR		(13)	3.58	4.85E-06
**LRRN2**			3.55	2.59E-04
CEACAM1	*		3.52	3.57E-06
IFI44L	*x		3.48	1.05E-06
**TMEM132E**			3.46	1.05E-03
IFIT2	*x		3.44	8.88E-03
**TAF5LP1**			3.41	2.84E-03
CTSS	*		3.38	6.61E-10
IFIH1	*x		3.38	8.17E-08
IFIT3	*x		3.37	4.34E-03
ANGPTL4	*		3.35	3.40E-08
IFI27	*x		3.35	8.65E-06
USP18	*x		3.33	6.20E-03
RIGI		(14)	3.33	1.11E-05
APOL4	*		3.32	2.48E-10
C3AR1		(15)	3.31	3.14E-04
ZMYND15	*		3.31	8.72E-11
STRA6		(16)	3.30	8.92E-04
THEMIS2	*		3.30	7.75E-06
OAS3	*x		3.30	1.54E-02
IFI44	*x		3.30	4.22E-06
IRF7	*x		3.29	1.88E-07
ISG20	*x		3.28	2.97E-10
CD274	*		3.25	3.53E-06
IL22RA1	*		3.23	6.85E-04
**TOMM20P2**			3.23	6.57E-03
FYB1		(17)	3.23	4.87E-05
EPSTI1	*x		3.22	3.15E-06
LRRTM2	*		3.22	5.85E-07
IFIT1	*x		3.22	4.81E-06
**KIAA1755**			3.22	8.20E-08
CD7	*		3.20	3.22E-05
ITK	*		3.20	1.92E-05
ETV7	*x		3.19	1.46E-06
AQP1		(18)	3.19	1.79E-07
GMPR	*x		3.19	3.32E-08
HELZ2	*		3.19	1.97E-05
**LGI2**			3.16	1.15E-04
APOBEC3G	*		3.15	3.27E-07
**PLEKHG4B**			3.15	3.72E-04
KIAA0040	*x		3.15	1.02E-03
SOCS1	*x		3.12	6.08E-07
LMO2	*x		3.12	2.20E-06

SAMD9L	*		3.08	3.84E-08
SUSD4	*		3.07	3.97E-04
CACNA1I	*		3.05	3.06E-05
**SHISAL1**			3.04	1.49E-06
LYPD5	*		3.03	1.60E-06
**NKAIN1**			3.03	1.24E-03
GBP1	*x		3.01	1.90E-06
TLR3	*x		3.00	1.35E-12
TGFA	*		3.00	1.08E-03
GSDMC	*		3.00	3.23E-05
IFI6	*x		3.00	4.56E-08
LINC02574		(19)	2.97	2.81E-07
SECTM1	*x		2.95	2.30E-12
TNFAIP8L3		(20)	2.94	5.02E-06
RTP4	*x		2.92	1.58E-07
HES4	*x		2.92	8.38E-10
IL15RA	*x		2.90	3.41E-15
PARP14	*		2.89	3.55E-10
LRRC4		(21)	2.88	1.97E-04
**SLCO5A1**			2.87	1.10E-04
CCL2	*		2.87	7.02E-07
ANO7L1	*		2.87	7.51E-13
TSPAN2	*		2.87	8.18E-06
TSPAN33	*		2.86	4.98E-05
FAM107A	*		2.86	1.38E-06
GCH1	*x		2.86	2.12E-06
RIMBP2			2.86	1.11E-04
PARP12	*x		2.85	2.60E-07
MSX1	*		2.84	8.69E-07
TAP1	*x		2.84	2.98E-08
PLSCR1	*x		2.84	1.65E-07
**SHC4**			2.83	1.58E-06
GPR68	*		2.82	4.39E-08
DDX60L		(22)	2.81	1.44E-07
SLC15A3	*x		2.81	2.41E-13
PGF		(23)	2.81	1.00E-04
CLDN23	*		2.81	7.67E-08
**LINC03033**			2.77	4.76E-04
**GDF10**			2.75	3.35E-04
C5AR2		(24)	2.74	7.44E-04
BATF3	*		2.72	6.79E-04
CASP1	*		2.71	2.57E-11
TRIM21	*x		2.70	8.21E-08

**MIR3918**			2.70	4.67E-03
MYD88	*x		2.68	6.18E-08
APOL2	*		2.67	7.46E-08
SP110	*x		2.65	1.57E-10
PSMB8-AS1		(25)	2.64	9.23E-10
SAMHD1	*x		2.60	5.13E-12
**PRAL**			2.60	3.40E-10
CD74	*		2.60	4.07E-04
IFI35	*		2.60	3.92E-09
PNPT1	*x		2.60	1.60E-07
SAMD9	*		2.60	8.06E-09
PSMB9	*x		2.58	5.24E-10
**ISLR2**			2.58	1.27E-03
APOL3	*		2.58	3.33E-15
RNF213	*		2.56	2.92E-08
APOBEC3F	*		2.56	5.42E-08
**FRMPD3**			2.54	1.02E-04
GIMAP2	*		2.54	4.13E-07
LINC02701		(26)	2.54	2.41E-03
TLDC2		(27)	2.54	1.24E-09
**FAM124A**			2.53	1.20E-03
SLC12A7	*		2.53	4.18E-11
**MYOM3**			2.53	1.11E-03
APOL1	*		2.52	1.71E-17
**NEBL**			2.52	3.03E-03
XAF1	*x		2.52	2.75E-10
MLKL	*		2.51	3.18E-07
HLA-DOB	*		2.49	4.62E-04
**IKBKGP1**			2.49	3.89E-03
SLC18B1	*		2.49	2.72E-09
DDX60	*		2.48	4.80E-12
RNF122	*		2.45	3.27E-10
**KCNA7**			2.44	1.59E-03
MAP2	*		2.44	1.72E-08
ACSL5	*		2.44	3.32E-08
TAP2	*x		2.43	2.26E-09
PLAAT4		(28)	2.43	5.66E-05
RASGRP3	*		2.43	2.06E-05
GRM4		(29)	2.42	2.38E-04
TYMP	*x		2.41	3.92E-12
**FIBCD1**			2.41	2.46E-03
**NKX3-1**			2.40	9.85E-06
GALNT3		(30)	2.40	7.06E-04

**HK2-DT**			2.39	4.67E-05
PARP9	*		2.37	5.78E-13
CALHM6		(31)	2.37	4.49E-03
SHFL		(32)	2.37	5.41E-11
TDRD7	*x		2.37	2.88E-07
TMEM229B	*		2.35	2.22E-07
KCTD14	*		2.34	1.60E-05
HEY1		(33)	2.34	6.99E-04
LAP3	*		2.34	1.52E-10
FGF18	*		2.33	1.35E-02
**CAVIN4**			2.33	3.18E-05
**AKAP5**			2.33	2.21E-03
**NKD1**			2.33	1.31E-02
ANGPTL1	*		2.32	4.70E-07
ANGPTL6	*		2.32	4.00E-11
NMI	*x		2.32	8.12E-09
**RNF213-AS1**			2.31	6.83E-08

References

1. Rusinova I, Forster S, Yu S, Kannan A, Masse M, Cumming H, et al. Interferome v2.0: an updated database of annotated interferon-regulated genes. Nucleic Acids Res. 2013;41(Database issue):D1040-6.
2. Schoggins JW, Wilson SJ, Panis M, Murphy MY, Jones CT, Bieniasz P, et al. A diverse range of gene products are effectors of the type I interferon antiviral response. Nature. 2011;472(7344):481-5.
3. Dissanayake TK, Schauble S, Mirhakkak MH, Wu WL, Ng AC, Yip CCY, et al. Comparative Transcriptomic Analysis of Rhinovirus and Influenza Virus Infection. Front Microbiol. 2020;11:1580.
4. Ahmad SM, Bhat SS, Shafi S, Dar MA, Saleem A, Haq Z, et al. Identification of key transcription factors and their functional role involved in Salmonella typhimurium infection in chicken using integrated transcriptome analysis and bioinformatics approach. BMC Genomics. 2023;24(1):214.
5. Li M, Zhang R, Li F, Wang H, Kim HJ, Becnel L, et al. Transfection of SSTR-1 and SSTR-2 inhibits Panc-1 cell proliferation and renders Panc-1 cells responsive to somatostatin analogue. J Am Coll Surg. 2005;201(4):571-8.
6. Wang H, Bi Z, Dai K, Li P, Huang R, Wu S, et al. A Functional Variant in the Aquaporin-3 Promoter Modulates Its Expression and Correlates With Resistance to Porcine Epidemic Virus Infection in Porcine Intestinal Epithelial Cells. Front Microbiol. 2022;13:877644.
7. Kiani A, Garcia-Cozar FJ, Habermann I, Laforsch S, Aebischer T, Ehninger G, et al. Regulation of interferon-gamma gene expression by nuclear factor of activated T cells. Blood. 2001;98(5):1480-8.
8. Tesser A, Piperno GM, Pin A, Piscianz E, Boz V, Benvenuti F, et al. Priming of the cGAS-STING-TBK1 Pathway Enhances LPS-Induced Release of Type I Interferons. Cells. 2021;10(4).
9. Mavrommatis E, Fish EN, Platanias LC. The schlafen family of proteins and their regulation by interferons. J Interferon Cytokine Res. 2013;33(4):206-10.
10. Woeckel VJ, Eijken M, van de Peppel J, Chiba H, van der Eerden BC, van Leeuwen JP. IFNbeta impairs extracellular matrix formation leading to inhibition of mineralization by effects in the early stage of human osteoblast differentiation. J Cell Physiol. 2012;227(6):2668-76.

11. Houston B, Stewart AJ, Farquharson C. PHOSPHO1-A novel phosphatase specifically expressed at sites of mineralisation in bone and cartilage. Bone. 2004;34(4):629-37.
12. Yuce K, Ozkan AI. The kruppel-like factor (KLF) family, diseases, and physiological events. Gene. 2024;895:148027.
13. Kambara H, Gunawardane L, Zebrowski E, Kostadinova L, Jobava R, Krokowski D, et al. Regulation of Interferon-Stimulated Gene BST2 by a lncRNA Transcribed from a Shared Bidirectional Promoter. Front Immunol. 2014;5:676.
14. Xu L, Wang W, Li Y, Zhou X, Yin Y, Wang Y, et al. RIG-I is a key antiviral interferon-stimulated gene against hepatitis E virus regardless of interferon production. Hepatology. 2017;65(6):1823-39.
15. Tripathi A, Whitehead C, Surrao K, Pillai A, Madeshiya A, Li Y, et al. Type 1 interferon mediates chronic stress-induced neuroinflammation and behavioral deficits via complement component 3-dependent pathway. Mol Psychiatry. 2021;26(7):3043-59.
16. He W, Sun Y, Ge J, Wang X, Lin B, Yu S, et al. STRA6 regulates tumor immune microenvironment and is a prognostic marker in BRAF-mutant papillary thyroid carcinoma. Front Endocrinol (Lausanne). 2023;14:1076640.
17. Rice S, Kim SM, Rodriguez C, Songock W, Raikhy G, Lopez R, et al. Suppression of a Subset of Interferon-Induced Genes by Human Papillomavirus Type 16 E7 via a Cyclin Dependent Kinase 8-Dependent Mechanism. Viruses. 2020;12(3).
18. Dicay MS, Hirota CL, Ronaghan NJ, Peplowski MA, Zaheer RS, Carati CA, et al. Interferon-gamma suppresses intestinal epithelial aquaporin-1 expression via Janus kinase and STAT3 activation. PLoS One. 2015;10(3):e0118713.
19. Zhang Y, Chi X, Hu J, Wang S, Zhao S, Mao Y, et al. LncRNA LINC02574 Inhibits Influenza A Virus Replication by Positively Regulating the Innate Immune Response. Int J Mol Sci. 2023;24(8).
20. Gu Z, Cui X, Sun P, Wang X. Regulatory Roles of Tumor Necrosis Factor-alpha-Induced Protein 8 Like-Protein 2 in Inflammation, Immunity and Cancers: A Review. Cancer Manag Res. 2020;12:12735-46.
21. Zhang Y, Li D, Zeng Q, Feng J, Fu H, Luo Z, et al. LRRC4 functions as a neuron-protective role in experimental autoimmune encephalomyelitis. Mol Med. 2021;27(1):44.
22. Grunvogel O, Esser-Nobis K, Reustle A, Schult P, Muller B, Metz P, et al. DDX60L Is an Interferon-Stimulated Gene Product Restricting Hepatitis C Virus Replication in Cell Culture. J Virol. 2015;89(20):10548-68.
23. Huang Q, Ding J, Gong M, Wei M, Zhao Q, Yang J. Effect of miR-30e regulating NK cell activities on immune tolerance of maternal-fetal interface by targeting PRF1. Biomed Pharmacother. 2019;109:1478-87.
24. Wright O, Harris A, Nguyen VD, Zhou Y, Durand M, Jayyaratnam A, et al. C5aR2 Regulates STING-Mediated Interferon Beta Production in Human Macrophages. Cells. 2023;12(23).
25. More S, Zhu Z, Lin K, Huang C, Pushparaj S, Liang Y, et al. Long non-coding RNA PSMB8-AS1 regulates influenza virus replication. RNA Biol. 2019;16(3):340-53.
26. Xu R, Yu SS, Yao RR, Tang RC, Liang JW, Pang X, et al. Interferon-Inducible LINC02605 Promotes Antiviral Innate Responses by Strengthening IRF3 Nuclear Translocation. Front Immunol. 2021;12:755512.
27. Hadifar S, Masoudzadeh N, Andersson B, Heydari H, Mashayekhi Goyonlo V, Kerachian M, et al. Integrated analysis of lncRNA and mRNA expression profiles in cutaneous leishmaniasis lesions caused by Leishmania tropica. Front Cell Infect Microbiol. 2024;14:1416925.
28. Zhao JY, Yuan XK, Luo RZ, Wang LX, Gu W, Yamane D, et al. Phospholipase A and acyltransferase 4/retinoic acid receptor responder 3 at the intersection of tumor suppression and pathogen restriction. Front Immunol. 2023;14:1107239.
29. Wan Z, Sun R, Liu YW, Li S, Sun J, Li J, et al. Targeting metabotropic glutamate receptor 4 for cancer immunotherapy. Sci Adv. 2021;7(50):eabj4226.
30. Guan Q, Ezzati P, Spicer V, Krokhin O, Wall D, Wilkins JA. Interferon gamma induced compositional changes in human bone marrow derived mesenchymal stem/stromal cells. Clin Proteomics. 2017;14:26.

31. Danielli S, Ma Z, Pantazi E, Kumar A, Demarco B, Fischer FA, et al. The ion channel CALHM6 controls bacterial infection-induced cellular cross-talk at the immunological synapse. EMBO J. 2023;42(7):e111450.
32. Suzuki Y, Murakawa T. Restriction of Flaviviruses by an Interferon-Stimulated Gene SHFL/C19orf66. Int J Mol Sci. 2022;23(20).
33. Hu X, Chung AY, Wu I, Foldi J, Chen J, Ji JD, et al. Integrated regulation of Toll-like receptor responses by Notch and interferon-gamma pathways. Immunity. 2008;29(5):691-703.

Supplementary Table 2. Up-regulated genes (log2(FC)>3.3 in ATAD3A, DGUOK, MPV17, PNPT1 and POLG-1 fibroblasts. ° and x and indicate genes that are up-regulated in control fibroblasts treated with interferon and known to be ISGs(1), respectively.
(1) Schoggins JW, Wilson SJ, Panis M, Murphy MY, Jones CT, Bieniasz P, et al. A diverse range of gene products are effectors of the type I interferon antiviral response. Nature. 2011;472(7344):481-5

ATAD3A

Symbol		LogFC	p-value
SMOC2		7.28582787440728	0.000007737839
PAX3		4.94520799157549	0.00259807
HHIP		4.38873165162051	0.00001582903
ESM1		4.28585813709411	0.0002729074
FGL2	°	4.1272433912588	0.005625626
ALDH1A1	x	3.6544615103445	0.00006128798
PALMD		3.61609926549319	0.0000061683
NTRK2		3.50633281440445	0.02652774
CRLF1		3.46583432261376	9.361099E-16
RPL10P9		3.30382116742132	3.462659E-47
ACAN		3.29537455152712	0.00000286048

DGUOK

Symbol		LogFC	p-value
CXCL8		6.51891878903622	0.000000000494614
H19		6.09083543898352	0.0000000005743723
PAX8-AS1		5.34921746644401	1.661734E-21
KLHDC7B		5.04533535292762	0.003424068
A2M		4.90165454180748	0.005707084
HSD11B1		4.87734622713137	0.0000002835125
HELLPAR		4.61887155598342	0.00000003597724
MSX2		4.35166107014497	0.004617594
C3		4.34734486427553	0.000001719113
CXCL1		4.27267170843978	0.000002373925
IL32		4.24519418249518	0.000002083661
PAX8		4.24221529650671	1.44682E-33
TNFAIP6	°x	4.19846062656737	0.0000001925739
CXCL6		4.08077191481138	0.003508397
RARRES2		4.01465463304479	0.00002556934
SBSPON		4.00870678794264	0.00004801083
PENK		3.96464506770635	0.000004408567
ZNF423		3.9581042841591	0.000000000003285007
CRLF1		3.88850752857905	5.239954E-15
G0S2		3.80376639606763	0.001145417
MEST		3.79649254504706	0.000000003833519
PDLIM3		3.78175102426946	0.008646248
CD36		3.68707748981474	0.001971899
ALPL		3.642446276545	0.008016829
SPON1		3.61762095277979	0.0000000008062397
CFB	x	3.61527189497134	0.0000000008189331
PENK-AS1		3.60404443107645	0.000007983861

IL18R1	3.51414204316182	0.01576232
SORBS2	3.49160001473603	0.00007629321
MGP	3.46136047972623	0.0008515493
RARRES1	3.45189684727843	0.000000000008994404
RUNX3 °	3.4264530756235	0.001255931
CCL2 °x	3.39747167885806	0.000001922511
ANKRD1	3.32635330841594	0.0000456692
INS-IGF2	3.30956281442351	0.002805053

MPV17

Symbol	LogFC	p-value
SOX11	6.05083352847914	0.0007669147
PAX3	5.29960391896693	0.001397202
PAX8-AS1	5.02066176109906	4.138632E-20
CORIN	4.8815856636999	0.000000210023
CPXM1	4.76363146778008	0.000000000002282486
CADM1	4.74456370203413	1.375262E-22
WFDC1	4.62035552167318	0.00000003354723
IGF2	4.38397403107139	0.005737675
INS-IGF2	4.37099747301109	0.005879464
CLEC14A	4.34367501594536	0.000000000006671088
RUNX3 °	4.26951246859658	0.00002520069
F2RL2	4.24586070184035	0.002643054
MGAT5B	4.22602729431835	0.00000000001718766
ZNF853	4.1426135713784	0.00000002199433
EXTL1	4.12284800694089	0.0000000105361
FGFR2	4.10475291234008	0.006693968
LRRN3	4.08688536479299	0.006834062
TRHDE	4.08588944599949	0.005851841
IQGAP2	4.03515466430753	0.002456895
NES	4.01808576035121	0.0000003807724
SIM2	3.95244788392802	3.967628E-16
MSX2	3.92421424494425	0.005082693
EFHD1	3.874349762753	0.00002041988
LZTS1	3.77431864567015	0.00005247665
MECOM	3.76714273776865	1.584769E-22
TRHDE-AS1	3.68105452233764	0.007070815
PLPPR3	3.62504288257653	0.0000001451355
DRP2	3.62182839191924	0.0002318365
PAX8	3.62133141501006	4.891661E-24
FILIP1	3.60243154117917	0.00001402903
ALDH1A1	3.59535128310879	0.0002868368
CITED4	3.53875376545236	8.691377E-15
AKAP6	3.49894268455856	0.0001794406
RASGRP1	3.48457856513256	5.911648E-18
NCAM1	3.43707101223511	0.000000000003783675
HMCN1	3.38120629748534	0.0000000765046
TGFB2-OT1	3.37321898109135	0.0000001329129

PTPRN		3.37220131125006	0.00000001503179
KCNH1		3.37136277285894	0.01006088
GABRA5		3.35408114221159	0.001360789
PNMA8A		3.33100003230187	0.00000001935108
TDRP		3.31109641616432	0.002755925
LINC01305		3.30972931829898	0.001095681
MARCHF9		3.30280946927445	0.0000001486672
PAX6		3.3022614297428	0.0000000005539839

PNPT1

Symbol		LogFC	p-value
CXCL8		7.96801316092837	1.207022E-18
CXCL1		6.28313911276553	7.048337E-15
CXCL6		6.26164890380002	0.0005021843
TNFAIP6	°x	6.00919800364102	1.980427E-26
PDPN		5.27192498986097	0.00000000116459
C3		4.97106648523076	0.00000003665805
MSX2		4.90469892063396	0.00152371
CYP7B1		4.86756325518884	0.0009288417
SFRP2		4.8630055826204	0.004567242
HSD11B1		4.50112934117436	0.00000095858
IL18R1		4.44030487701445	0.003074577
CFB	x	4.3658873000108	9.8174E-18
RIPOR3		4.11520779670983	0.005905663
CPXM1		4.09574132953678	0.0000002838449
CCL2	°x	3.99248747661567	0.000000000000592563
MMP1		3.98171020294943	0.0001423511
WTAPP1		3.85744427908013	0.0001713066
LSP1		3.8432582954658	0.0006595553
KLHDC7B		3.68979451262032	0.006643789
H19		3.66906494954287	0.0001519508
IL1RL1	°	3.5824998034482	0.01372253
IL6-AS1		3.47431575956925	0.0003439716
RUNX3	°	3.46091595631568	0.0007372206
XPNPEP2		3.35916879345634	0.0001903849
SLC39A8		3.33307065006893	0.0002619162
SOD2		3.33128100064756	0.000000007839096

POLG-1

Symbol	LogFC	p-value
CPXM1	5.76727933443	1.065687E-15
SMOC2	5.54610392419285	0.0004418583
MSX2	5.51377885577939	0.0008148584
PAX3	5.23675217809077	0.001885745
H19	4.94018351133761	0.000000000144523
PAX8-AS1	4.75238269838438	1.67402E-18
PCSK9	4.22683808221923	0.000002508047
IL18R1	4.17516341639249	0.004843535

GPX1P1		3.94939703777486	3.624951E-38
RPL10P6		3.64102033609068	1.132196E-36
INS-IGF2		3.57676236554233	0.000953856
RIPOR3		3.56860331188869	0.009512342
ADH1B		3.56062243970758	0.01503558
PAX8		3.55887414062844	8.287616E-27
ENPP5		3.5473618070898	0.00004993229
STEAP4	°x	3.52429022866257	0.004227497
IGF2		3.49720144662993	0.0006731413
BCHE		3.35914817298932	0.01170629

Supplementary Table 3. Oligonucleotides for mtDNA quantification and TaqMan probes for ISG quantification.

TaqMan probes for mtDNA quantification

Target	RNA/DNA	Reference
RNA18SN1	DNA	Hs99999901_s1
MT-RNR2	DNA	Hs02596860_s1
MT-CO1 I	DNA	Hs02596864_g1

TaqMan probes for ISG quantification

Target	RNA/DNA	Reference
IFI44L	RNA	Hs00199115_m1
IFI27	RNA	Hs01086370_m1
RSAD2	RNA	Hs00369813_m1
IFIT1	RNA	Hs01675197_m1
ISG15	RNA	Hs01921425_s1
IFNB1	RNA	Hs01077958_s1
GAPDH	DNA	Hs02786624_g
